# Podocyte Injury Associated with Mutant ****α****-Actinin-4

**DOI:** 10.1155/2011/563128

**Published:** 2011-07-26

**Authors:** Andrey V. Cybulsky, Chris R. J. Kennedy

**Affiliations:** ^1^Department of Medicine, McGill University Health Centre, McGill University, Montreal, QC, H3A 1A1, Canada; ^2^Division of Nephrology, Royal Victoria Hospital, 687 Pine Avenue West, Montreal, QC, H3A 1A1, Canada; ^3^Kidney Research Centre, Department of Medicine, The Ottawa Hospital, University of Ottawa, Ottawa, ON, Canada K1H 8M5; ^4^Departments of Medicine and CMM, Ottawa Hospital Research Institute, University of Ottawa, 451 Smyth Road, Ottawa, ON, K1H 8M5, Canada

## Abstract

Focal segmental glomerulosclerosis (FSGS) is an important cause of proteinuria and nephrotic syndrome in humans. The pathogenesis of FSGS may be associated with glomerular visceral epithelial cell (GEC; podocyte) injury, leading to apoptosis, detachment, and “podocytopenia”, followed by glomerulosclerosis. Mutations in **α**-actinin-4 are associated with FSGS in humans. In cultured GECs, **α**-actinin-4 mediates adhesion and cytoskeletal dynamics. FSGS-associated **α**-actinin-4 mutants show increased binding to actin filaments, compared with the wild-type protein. Expression of an **α**-actinin-4 mutant in mouse podocytes *in vivo* resulted in proteinuric FSGS. GECs that express mutant **α**-actinin-4 show defective spreading and motility, and such abnormalities could alter the mechanical properties of the podocyte, contribute to cytoskeletal disruption, and lead to injury. The potential for mutant **α**-actinin-4 to injure podocytes is also suggested by the characteristics of this mutant protein to form microaggregates, undergo ubiquitination, impair the ubiquitin-proteasome system, enhance endoplasmic reticulum stress, and exacerbate apoptosis.

## 1. Introduction

Glomerular visceral epithelial cells (GECs; podocytes) play a key role in the maintenance of glomerular permselectivity [1–4]. Under normal conditions, podocytes are in contact with extracellular matrix, and there appears to be little turnover of podocytes, as these cells are highly differentiated (“terminally differentiated”). GECs form a tight network of interdigitating foot processes, which are bridged by filtration-slit diaphragms, and permselectivity of the glomerular capillary wall is dependent on the maintenance of appropriate structure of podocytes and of their foot processes. The complex cellular architecture of the podocyte is maintained by an organization of actin filaments in the cytoplasm. Proteins including nephrin, neph1, FAT, podocin, CD2AP and ZO-1 are found within or near the slit diaphragm, and nephrin, neph family proteins, and cadherins form complexes with scaffolding proteins, CD2AP and ZO-1 [2, 4]. These connect the slit-diaphragm protein complex with actin filaments, which are joined by *α*-actinin-4 proteins.

Acquired GEC injury is frequently associated with effacement of the foot processes, disruption of the filtration-slit diaphragms, and proteinuria [1, 2]. Moreover, heritable mutations in several GEC structural proteins alter podocyte ultrastructure and cause heritable proteinuria, implying that impairment of the slit diaphragm or cytoskeletal structure underlies proteinuria [4, 5]. Focal segmental glomerulosclerosis (FSGS) is an important cause of proteinuria and nephrotic syndrome in humans. In recent years, evidence has accumulated to support the view that the early pathogenesis of FSGS is associated with GEC injury, leading to apoptosis, detachment of GECs from extracellular matrix, and “podocytopenia”, which is followed by glomerulosclerosis [1–3, 6–8]. Acquired GEC injury may be triggered by immunological factors (e.g., T cell or other factors affecting permeability), oxidants, human immunodeficiency virus, toxins, and other substances. In addition, FSGS may be associated with heritable mutations in several distinct proteins that play key roles in maintaining GEC ultrastructure, including podocin, *α*-actinin-4, transient receptor potential cation channel, subfamily C, member 6 (TRPC6), inverted formin 2 and others [5]. In this paper, we focus on mutations in *α*-actinin-4, which are associated with an autosomal dominant late-onset FSGS in humans [9]. We discuss the structure and function of *α*-actinin-4, and we review mechanisms by which mutations in *α*-actinin-4 may lead to podocyte injury and FSGS.

## 2. Role of ***α***-Actinin-4 in Cell Biology

### 2.1. Structure of *α*-Actinin-4

Among the various cytoskeletal proteins, *α*-actinin-4 plays an important role in podocyte biology. There are four *α*-actinin genes (*ACTN1-4*) that encode highly homologous proteins (*α*-actinin-1, 2, 3 and 4), which form ~100 kDa head-to-tail homodimers [10]. *α*-actinins-2 and -3 are Ca^2+^-insensitive muscle *α*-actinins expressed in Z-discs of striated muscle cells [11], while *α*-actinins-1 and -4 are widely distributed, nonmuscle isoforms [12]. All family members are actin crosslinking proteins that share a number of structural features and regulatory regions. These include a central rod with four spectrin-like repeats, two N-terminal calponin-homology domains (CH1 and CH2), which contain three actin-binding sites (ABS1-3), a C-terminal calmodulin-like domain, containing two putative EF hands, and a phosphoinositide-binding plextrin homology domain. The spectrin-like repeats facilitate homodimerization and may confer a degree of flexibility to the *α*-actinin molecule, allowing it to resist mechanical strain [13]. For its interaction with actin filaments, the CH1 and CH2 domains adopt a closed conformation, where ABS 2 and 3 are exposed, while ABS1 remains buried and inaccessible to F-actin [14]. This association with actin is highly dynamic and can be controlled by the regulatory regions along the *α*-actinin sequence. 

### 2.2. Regulation and Function of *α*-Actinin-4

All *α*-actinin isoforms contain two putative EF-hand motifs at the C-terminus of each monomer, but only isoforms 1 and 4 demonstrate sensitivity to Ca^2+^ [15, 16]. For these nonmuscle isoforms, binding of Ca^2+^ is believed to reduce the affinity of *α*-actinin for actin, providing a mechanism for *α*-actinin-dependent cytoskeletal remodeling. Additionally, extensive biochemical studies on the effect of phosphoinositide binding to *α*-actinin-1 have been performed. Binding of phosphatidylinositol (3,4,5)-trisphosphate (PIP_3_), and to a lesser extent phosphatidylinositol (4,5)-bisphosphate (PIP_2_), decreases the association between *α*-actinin-1 and F-actin, suggesting that phosphoinositides mediate cytoskeletal remodeling [17, 18]. For *α*-actinin-4, the phosphoinositide-binding site is within the CH2 domain. In contrast to findings for *α*-actinin-1, our studies showed that phosphoinositides increase the interaction of *α*-actinin-4 with F-actin [19]. Phosphorylation of *α*-actinin-1 has been reported in activated platelets [20]. Tyrosine phosphorylation at position 12 was found to be dependent on focal adhesion kinase (FAK), supporting a role for *α*-actinin in cell adhesion. Phosphorylation of *α*-actinin-1 reduced its association with F-actin *in vitro*, suggesting that *α*-actinin mediates FAK-dependent cytoskeletal remodeling [21]. The amino acid sequence of *α*-actinin-4 reveals an analogous tyrosine residue at position 32, suggesting that phosphorylation may also regulate the binding of *α*-actinin-4 to F-actin. However, we were unable to demonstrate FAK-dependent tyrosine phosphorylation of *α*-actinin-4 (unpublished observations). These findings suggest that regulation of *α*-actinin-1 and 4 are not identical.

Nonmuscle *α*-actinins are often regarded as simple actin filament crosslinking proteins. Expression of these isoforms at the cytoplasmic face of several types of cellular interaction sites, including focal adhesions, adherens junctions, and hemidesmosomes suggests a degree of versatility. The first *α*-actinin binding partners identified were the *β* subunits of integrins and intercellular adhesion molecule-1 [12]. Interactions between *α*-actinin and adhesion receptors may provide structural stability for the adhesion sites. Since *α*-actinins also bind to various regulatory molecules, *α*-actinin may also serve as a scaffolding protein to integrate signaling molecules at various adhesion sites. *α*-actinin-1 may link membrane proteins such as talin, vinculin, and *β*-integrins with the cortical actin cytoskeleton [10, 22–25]. Similarly, the tight junction protein, MAGI-1, interacts with the C-terminus of *α*-actinin-4, providing a physical link from the cell periphery to the cytoskeleton [26]. The list of interacting proteins is likely to grow and will provide a better understanding of the diverse functions of *α*-actinins in nonmuscle tissues. However, the best defined function of *α*-actinin-4 is to crosslink/bundle actin filaments, and it may enhance cell motility and elicit tumor-suppressor activity [10]. 

Intriguingly, *α*-actinin-4 is reported to shuttle between the cytoplasm and the nucleus. Cytoplasmic localization was associated with an infiltrative phenotype and correlated significantly with a poorer prognosis in cases of breast cancer [10]. In this context, the relevance of nuclear *α*-actinin-4 remains unknown, although it has been suggested that nuclear translocation may attenuate metastatic potential. The nuclear localization of *α*-actinin-4 suggests a role for *α*-actinin-4 in gene expression. *α*-actinins-1 and -4 were identified as histone deacetylase 7 (HDAC7)-interacting proteins, and the interaction domain was mapped to the C-terminus of *α*-actinin 4 [27]. HDAC7 can participate in multiple cellular processes, including the regulation of myocyte enhancer factor-2- (MEF2-) mediated transcription. A point mutation in HDAC7 disrupted its association with *α*-actinin-4 and MEF2, implying that *α*-actinin-4 and MEF2 binding sites overlap. Ectopic expression of *α*-actinin-4 disrupted the HDAC7-MEF2A association, and enhanced MEF2-mediated transcription. Overexpression of *α*-actinin-4 also potentiated estrogen receptor *α*-mediated transcription by counteracting a negative regulatory effect of HDAC7, while knockdown of *α*-actinin-4 decreased expression of estrogen receptor *α* target genes and affected proliferation of cultured breast cancer cells [28]. Another study demonstrated that *α*-actinin-4 colocalized along actin stress fibers and in membrane lamellae with nuclear factor-*κ*B (NF-*κ*B) [29]. Incubation of cells with epidermal growth factor or tumor necrosis factor-*α* induced translocation of *α*-actinin-4 and the p65 subunit of NF-*κ*B into the nucleus. Moreover, *α*-actinin-4 and the p65 underwent association. As NF-*κ*B regulates transcription of a large number of genes in response to diverse stimuli, the study further supports a regulatory role for *α*-actinin-4 in transcription.

## 3. ***α***-Actinin-4 in Podocyte Biology

### 3.1. *α*-Actinin-4 in the Normal Podocyte

**Figure 1:**
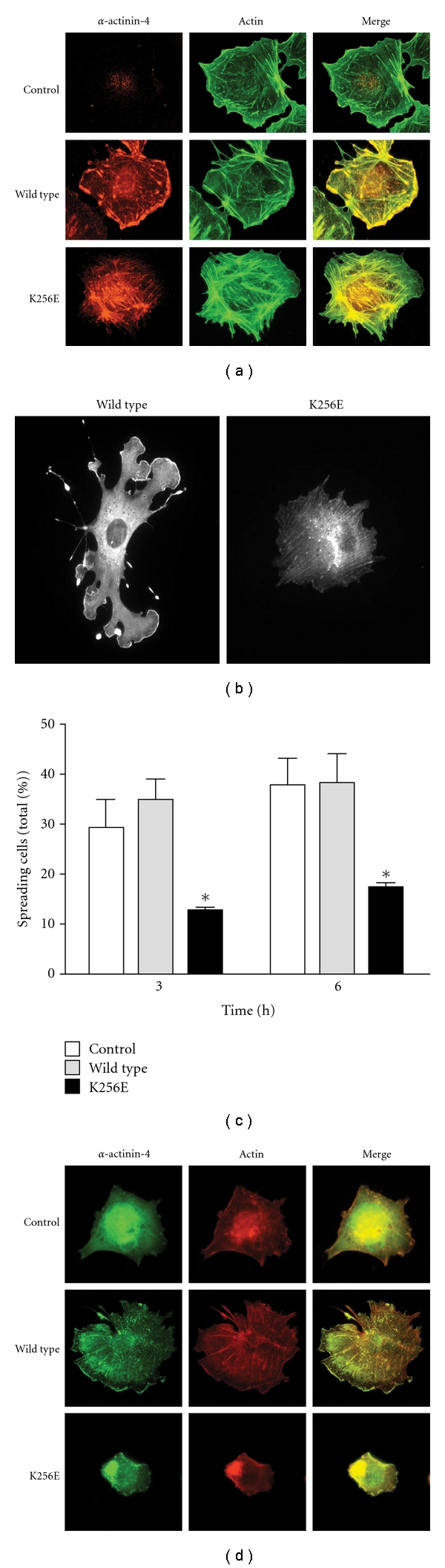
Subcellular localization of wild-type and K256E *α*-actinin-4 in mouse podocytes and effects on cell spreading. (a) Differentiated podocytes were infected with adenoviruses encoding HA-tagged *α*-actinin-4 K256E and wild type. Cells were fixed and stained with an anti-HA antibody to detect *α*-actinin-4, and phalloidin to detect F-actin. Uninfected cells were treated in the same manner and serve as control. Wild-type *α*-actinin-4 is predominantly found with membrane-associated cortical actin and process-like projections with limited expression along stress fibers. Conversely, *α*-actinin-4 K256E is predominantly associated with stress fibers and is excluded from the cell periphery. (b) A differentiated mouse podocyte expressing GFP-tagged wild-type or K256E *α*-actinin-4. The wild type expressing cell forms numerous *α*-actinin-4-containing lamellipodia in response to serum stimulation. While *α*-actinin-4 K256E readily decorates actin stress fibers, it, nevertheless, forms large aggregates, and the cells fail to produce lamellipodia. (c, d) Differentiated mouse podocytes expressing GFP alone (control), wild-type, or K256E *α*-actinin-4 were trypsinized, held in suspension for 15 min, replated onto collagen I-coated coverslips, and analyzed by fluorescence microscopy at 3 and 6 h. Expression of *α*-actinin-4 K256E significantly reduced the number of spreading cells. After 6 h, wild-type *α*-actinin-4 was localized to cortical actin, whereas K256E *α*-actinin-4 remained condensed within the cell. (b) *P < .05 versus control and wild-type of corresponding time point. Panels (a), (c), and (d) of the figure are adapted from [33] with permission from the Nature Publishing Group.

Human GECs express only *α*-actinin-4 (although mouse GECs also express *α*-actinin-1) [10, 30]. Immunoelectron microscopy studies showed that *α*-actinin localizes to contractile microfilaments within podocyte foot processes [31, 32]. In cultured mouse GECs, *α*-actinin-4 was found along actin stress fibers, focal adhesions, and within the cortical actin network at the cell periphery (Figure 1) [33]. Analogous findings were evident in rat GECs [34, 35]. This specific subcellular localization of *α*-actinin-4 facilitates its regulation of adhesion and cytoskeletal dynamics [19, 33, 36]. Interestingly, *α*-actinin-4 was also present in the nucleus of some rat GECs [34, 35], in keeping with earlier reports in other cell lines [10]. Normally, *α*-actinin-4 may be required for integrin-dependent adhesion of GECs [36]. Indeed, knockout of *α*-actinin-4 in mice resulted in reduced glomerular podocyte number, accompanied by the appearance of urinary Wilm's tumor-1 (WT-1), a podocyte biomarker. Such podocyte loss is consistent with defective glomerular basement membrane adhesion, as TUNEL assays failed to detect any apoptotic cells in *α*-actinin-4 null glomeruli. The severity of the resulting phenotype seen in *α*-actinin-4 null mice (i.e., glomerular disease, renal failure, and early death) indicates that *α*-actinin-4-mediated podocyte adhesion is critical for filtration barrier function [30, 37, 38]. On the other hand, transgenic podocyte overexpression of wild-type *α*-actinin-4 in mice did not alter glomerular filtration barrier function [39]. These findings suggest that podocytes tolerate a wide range of *α*-actinin-4 expression, but that a minimum threshold level is essential for normal GEC adhesion to the glomerular basement membrane through its interaction with integrins. 

### 3.2. Effects of Mutations in *α*-Actinin-4 on Podocyte Cytoskeletal Structure and Function

Deficiency of *α*-actinin-4 leading to human disease is not described, but several point mutations or single amino acid deletions in *α*-actinin-4 are found in heritable forms of human FSGS. Most mutations in *ACTN4*-associated FSGS families congregate at the CH1-CH2 interface [9, 40]. An example is the autosomal dominant K255E mutation (K256E is the mouse mutant corresponding to human K255E). Such mutants show increased binding to actin filaments, compared with the wild-type protein [30, 41, 42]. Interestingly, *α*-actinin-4 K256E is insensitive to regulation by Ca^2+^ and phosphoinositides (PIP_2_ and PIP_3_), suggesting that its gain of affinity for F-actin blunts its responsiveness to regulatory factors [19]. Expression of an *α*-actinin-4 K256E transgene in mouse podocytes *in vivo* (under the control of the nephrin promoter) resulted in development of proteinuric FSGS [41]. In addition, homozygous (but not heterozygous) “knock-in” of the mutant *α*-actinin-4 allele into the *ACTN4* locus in mice induced proteinuria [30], confirming the pathogenic potential of *α*-actinin-4 mutations. 

The underlying biochemical mechanism, whereby FSGS-associated *ACTN4* mutations enhance affinity for actin, was uncovered by Pollak's group. They showed that disease-causing mutations disable an important intramolecular hinge that normally holds CH1 and CH2 in a “closed” conformation [43]. The mutant protein adopts an open conformation forcing an interaction of all three actin-binding sites with the actin filament, thereby increasing the binding affinity by lowering its rate of dissociation from actin [44]. 

## 4. How Do the Biophysical Effects of Mutant ***α***-Actinin-4 Translate into a Dysfunctional Cellular Phenotype?

**Figure 2:**
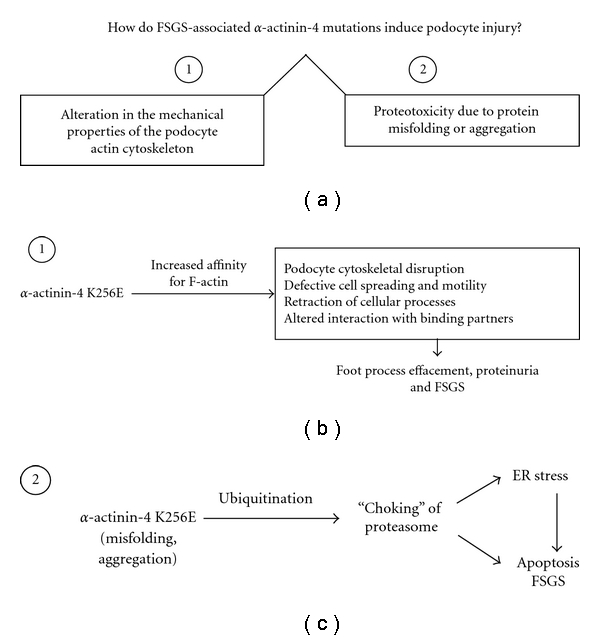
Podocyte injury induced by mutant *α*-actinin-4. (a) Potential actions of *α*-actinin-4 K256E and other FSGS-associated mutants. (b, c) Potential mechanisms for the cytoskeletal (b) and proteotoxic (c) effects of mutant *α*-actinin-4 in the podocyte.

Although *α*-actinin-4 is widely expressed, human podocytes appear to be selectively vulnerable to mutations in *α*-actinin-4 (including K256E and other analogous mutants), perhaps because podocytes are “terminally differentiated” cells with limited regenerative capacity. The downstream cellular mechanism(s) by which mutant *α*-actinin-4 leads to podocyte injury and FSGS is poorly understood. One possibility is that injury is secondary to alterations in the mechanical properties of the podocyte via increased affinity of mutant *α*-actinin-4 for F-actin (Figure 2). GECs that express *α*-actinin-4 K256E show defective spreading and motility (Figure 1) [33]. Moreover, exposure of *α*-actinin-4 K256E expressing GECs to cyclical equibiaxial stretch, an *in vitro* mimic of the mechanical forces due to glomerular capillary pressure, significantly reduced cell surface area and caused retraction of cellular processes [19]. Lastly, the enhanced association with F-actin alters the subcellular localization of *α*-actinin-4, restricting its presence at the cell periphery (Figure 1), and potentially altering its capacity to interact with various binding partners [19, 33]. Such abnormalities could, at least in part, be attributable to cytoskeletal disruption, as well as loss of integrin engagement, effectively yielding a phenotype similar to that of the *α*-actinin-4 null mouse [36, 43]. Since the actin cytoskeleton is a key component contributing to the unique morphological characteristics of the podocyte foot processes, as well as being connected to both slit diaphragm and adhesion complexes, altered *α*-actinin-4 biology must have important implications for podocyte health. 

An alternate and/or parallel mechanism, which we also consider in this paper, is that mutant *α*-actinin-4 induces proteotoxicity in podocytes (i.e., an impairment of podocyte function caused by misfolding of a protein), ultimately leading to apoptosis (Figure 2). Consistent with this notion, *α*-actinin-4 K256E forms actin-associated aggregates in cultured GECs and in podocytes of both homozygous K256E “knock-in” mice and humans with *ACTN4*-associated FSGS [30, 33, 35]. Such misfolded/aggregated protein may be associated with the activation of stress pathways in podocytes (see below). 

## 5. The Ubiquitin-Proteasome System and Endoplasmic Reticulum (ER) Stress

Prior to discussing the proteotoxic potential of mutant *α*-actinin-4, this section provides a brief overview of the ubiquitin-proteasome system and ER stress. The ubiquitin-proteasome system plays a key role in regulating the half-life of short-lived cellular proteins, and in selective degradation of damaged or abnormal proteins [45, 46]. The proteasome degrades aberrant cytoplasmic or cytoskeletal proteins, and misfolded ER proteins, which are retrotranslocated selectively to the cytoplasm. The latter process is known as ER-associated degradation (ERAD) [47, 48]. Ubiquitin-proteasome pathway-mediated protein degradation involves tagging of the substrate by covalent attachment of ubiquitin molecules via a three-step reaction, and degradation of the tagged protein by the 26S proteasome complex. Ubiquitinated proteins typically undergo efficient degradation by the proteasome, but sometimes, large amounts of misfolded proteins are not degraded effectively, and may form covalently-linked aggregates. Such misfolded proteins and/or aggregates may impair the function of the proteasome and lead to the activation of stress pathways and cytotoxicity [45].

Secretory, luminal, and membrane proteins normally attain their correctly folded conformation in the ER via ER-resident chaperones. To rescue misfolded proteins, the ER has in place quality control machinery, including the unfolded protein response (UPR) [49–53], and ERAD [47, 48]. In the UPR, accumulation of misfolded proteins in the ER results in the activation of three ER “sensors”. Activating transcription factor-6 moves from the ER to the Golgi, where it is cleaved by proteases. The cleaved cytosolic fragment translocates to the nucleus to activate transcription of ER chaperones, for example, the glucose-regulated proteins (grp), grp94 and bip (grp78), and others. In parallel, inositol requiring-1*α* activates its endoribonuclease activity, cleaving X-box binding protein-1 mRNA and changing the reading frame to yield a potent transcriptional activator. Normally, ER chaperones assist in posttranslational processing of proteins and in their exit from the ER, and may complex with defective proteins to target them for degradation. During stress, induction of ER chaperones may enhance the protein folding capacity, and limit accumulation of abnormal proteins. 

Misfolded proteins in the ER also activate PERK (PKR-like ER kinase), which then phosphorylates the eukaryotic translation initiation factor-2*α* subunit (eIF2*α*). This process reduces initiation AUG codon recognition, and the general rate of translation is blunted (which decreases the protein load on a damaged ER). The UPR allows cells to recover from stress, and may be protective to additional insults, but substantial/prolonged ER stress may lead to apoptosis. For example, certain mRNAs may be translated preferentially after eIF2*α* is phosphorylated. Among these is activating transcription factor-4, which induces expression of several genes, including CHOP (C/EBP homologous protein-10; also known as GADD153), a gene closely associated with apoptosis and/or growth arrest [49, 51]. Apoptosis may also result from activation of caspase-12 or protein kinases [49, 51]. Impairment of the ubiquitin-proteasome system can be associated with exacerbation of ER stress [49, 54], perhaps by interference with ERAD. 

## 6. Evidence for the Proteotoxicity of ***α***-Actinin-4 K256E

The potential for mutant *α*-actinin-4 to impair podocyte function is suggested by the characteristics of this mutant protein to form microaggregates, undergo ubiquitination, impair the ubiquitin-proteasome system, enhance ER stress, and enhance apoptosis (Figure 2).

### 6.1. *α*-Actinin-4 K256E Forms Microaggregates

Density-gradient centrifugation of the K255E mutant *α*-actinin-4 showed abnormal sedimentation, suggesting that the mutant protein forms high molecular mass aggregates [30]. In keeping with this result, expression of *α*-actinin-4 K255E (and other FSGS-inducing mutants), but not wild-type *α*-actinin-4 in cultured mouse GECs resulted in formation of aggregates, as visualized by fluorescence microscopy [30, 33, 42, 43]. Analogous results were obtained in rat GECs stably transfected with *α*-actinin-4 K256E, where in some cells, the mutant (but not the wild type) protein formed aggregates [35]. Unlike the wild-type protein, the mutant was not present in the nucleus of rat GECs [34, 35]. In addition, *α*-actinin-4 K255E formed aggregates in podocytes of homozygous K255E “knock-in” mice and humans with *ACTN4*-associated FSGS [30]. Misfolded/aggregated proteins may result in the activation of stress pathways [45]. 

### 6.2. *α*-Actinin-4 K256E Undergoes Ubiquitination and Proteasomal Degradation

**Figure 3:**
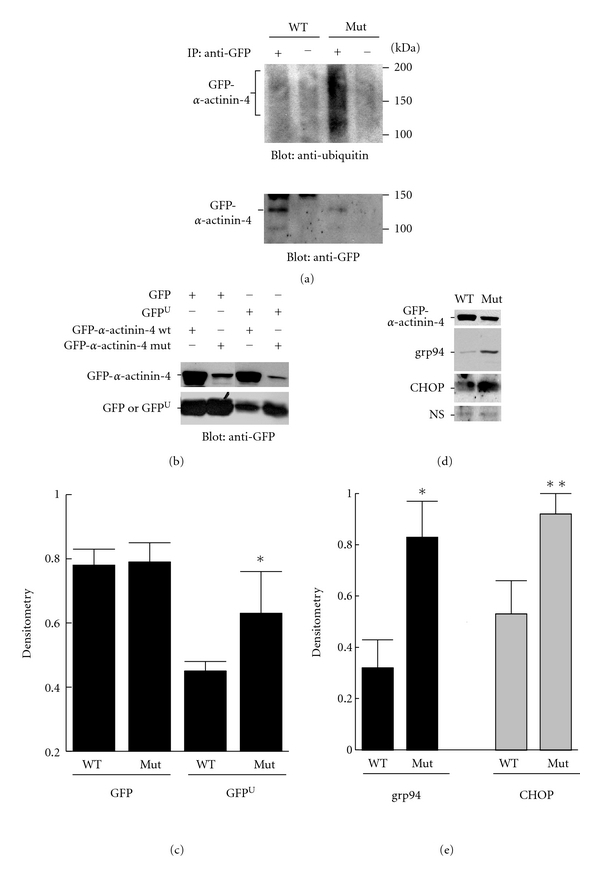
*α*-actinin-4 K256E “chokes” the proteasome and exacerbates ER stress. (a) GFP-*α*-actinin-4 K256E undergoes ubiquitination. COS-1 cells were transiently transfected with GFP-*α*-actinin-4 wild type (WT) or K256E mutant (Mut), and the cells were incubated with the proteasome inhibitor, MG132. Lysates were immunoprecipitated with anti-GFP antibody (+), or nonimmune IgG (−) in controls. Then, the immunoprecipitates were immunoblotted with antibodies to ubiquitin (upper panel) or GFP (lower panel). (b, c) COS cells were transiently cotransfected with the GFP^U^ proteasome reporter (or GFP for comparison), plus GFP-*α*-actinin-4 K256E or wild type. Lysates were immunoblotted with anti-GFP antibody. In COS cells transfected with *α*-actinin-4 wild type, expression of the GFP^U^ reporter is substantially lower than GFP, since GFP^U^ is readily degraded by the ubiquitin-proteasome pathway, but GFP is stable. Mutant *α*-actinin-4 did not affect GFP expression. GFP^U^ expression was enhanced by cotransfection of *α*-actinin-4 K256E, indicating that this mutant impaired degradation of GFP^U^ by the ubiquitin-proteasome system. (c) *P < .035 GFP^U^ mutant versus wild type. (d, e) COS cells were transiently transfected with GFP-*α*-actinin-4 wild type or mutant. Lysates were immunoblotted with antibodies to GFP, grp94 or CHOP. NS, nonspecific band-loading control. *P < .03, **P < .045 mutant versus wild type. The figure is adapted from [35] with permission of the American Physiological Society.

In pulse-chase metabolic labeling experiments, mutant *α*-actinin-4 was degraded more rapidly, compared with the wild-type protein, with the mutant showing a half-life of ~15 h, and the wild type of over 30 h [30]. Consistent with this result, after stable transfection in rat GECs or transient transfection in COS cells (a monkey kidney cell line, which allows for high levels of protein expression), the level of *α*-actinin-4 K256E protein was considerably lower, compared with the wild-type protein (Figure 3) [34, 35]. Together, these results suggested that *α*-actinin-4 K256E may undergo ubiquitination prior to degradation via the ubiquitin-proteasome pathway. Indeed, in transiently transfected COS cells, mutant *α*-actinin-4 was polyubiquitinated, whereas the wild-type protein was not (Figure 3) [35]. Treatment of GECs that express *α*-actinin-4 K256E with proteasome inhibitors enhanced expression of *α*-actinin-4 K256E, in keeping with ubiquitination and degradation of the mutant protein by the proteasome [30, 35]. 

### 6.3. *α*-Actinin-4 K256E Impairs the Ubiquitin-Proteasome Pathway

*α*-actinin-4 K256E and wild-type proteins were transiently overexpressed in COS cells to study their effects on the ubiquitin-proteasome system. The function of the ubiquitin-proteasome system was monitored in viable cells by use of a reporter consisting of a short degron, CL1, fused to the C-terminus of green fluorescent protein (GFP^U^) [55]. This GFP^U^ proteasome reporter is rapidly degraded when ubiquitin-proteasome function is normal, whereas impairment of the ubiquitin-proteasome system, for example, via enhanced flux of a mutant/aggregated protein will reduce degradation of GFP^U^, resulting in an increased level of GFP^U^ expression. In COS cells transfected with wild-type *α*-actinin-4, expression of GFP^U^ declined significantly, in keeping with proteasomal degradation of GFP^U^ (Figure 3). In contrast, expression of *α*-actinin-4 K256E retarded the degradation of GFP^U^, implying that the ubiquitin-proteasome system was impaired in the presence of mutant *α*-actinin-4 [35]. 

### 6.4. *α*-Actinin-4 K256E Enhances ER Stress

*α*-actinin-4 is a cytosolic/cytoskeletal protein that normally would not enter the ER to undergo posttranslational modification. Nonetheless, compared with *α*-actinin-4 wild type, transient transfection of *α*-actinin-4 K256E in COS cells enhanced the UPR, as evidenced by increased expression of the ER chaperone, grp94, and the proapoptotic gene, CHOP (Figure 3) [35]. In GECs, expression of the K256E mutant or wild type *α*-actinin-4 (by stable transfection) did not increase expression of the ER chaperone, bip, or CHOP, compared with parental (untransfected) cells. This difference between COS cells and GECs may be due to lower expression of stably transfected proteins in the GECs. However, after incubation of GECs with tunicamycin (a drug that blocks N-linked glycosylation and causes an accumulation of misfolded proteins in the ER [51]), the GECs stably transfected with *α*-actinin-4 K256E showed increases in bip and CHOP that significantly exceeded the increases seen in cells expressing the wild-type protein, as well as the increases in parental GECs [35]. The effect of *α*-actinin-4 K256E on the induction of the UPR by tunicamycin was particularly robust, given that expression of the mutant was substantially lower than the wild-type protein. Thus, although stable expression of *α*-actinin-4 K256E in GECs did not induce the UPR directly, the mutant appeared to adversely affect the integrity of the ER, which may render these cells more susceptible to additional stress and induction of proapoptotic genes. Together, these results are in keeping with the view that exacerbation of ER stress may be secondary to impairment of the ubiquitin-proteasome system [49, 54].

### 6.5. Mutant *α*-Actinin-4 Affects Apoptosis/Cell Survival

Stable expression of *α*-actinin-4 K256E in GECs led to a reduction in cell number, as well as increased apoptosis and caspase-3 activity, compared with GECs that stably express *α*-actinin-4 wild type, or parental (untransfected) GECs [34]. These changes in cell number and apoptosis in the presence of *α*-actinin-4 K256E occurred despite the significantly lower expression level of the mutant, compared with the wild-type *α*-actinin-4, highlighting the potential detrimental consequences of this mutation. In COS cells, *α*-actinin-4 K256E had only a slight effect on apoptosis and cell number, suggesting that this cell type may be more resistant to cytotoxicity. Nevertheless, *α*-actinin-4 K256E markedly exacerbated apoptosis and reduced cell number in the context of mild proteasome inhibition [35]. This result supports the view that apoptosis induced by mutant *α*-actinin-4 may be associated with an impairment in proteasome function.

### 6.6. ER Stress Is Evident in *α*-Actinin-4 K256E-Associated Podocyte Injury

The effects of *α*-actinin-4 K256E on the UPR (discussed above) are based on studies in cultured cell lines, but importantly, analogous changes also occurred *in vivo*. As stated above, transgenic mice that express an *α*-actinin-4 K256E transgene in podocytes develop proteinuria and FSGS [41]. Glomeruli were isolated from these mice and examined for evidence of ER stress [35]. Expression of the ER chaperones, bip and grp94, eIF2*α* phosphorylation, as well as expression of the proapoptotic protein, CHOP, were increased in glomeruli of transgenic mice, compared with control. Based on these results, it is reasonable to propose that in the *α*-actinin-4 K256E model of FSGS, there is pronounced ER stress, which may be contributing, at least in part, to GEC apoptosis.

## 7. Conclusion

The maintenance of a highly dynamic actin-based cytoskeleton is critically important to podocyte morphology and function. Microfilaments in the foot processes tether the actin cytoskeleton to the slit diaphragm and adhesion complexes, while forming the architectural infrastructure for the foot processes. *α*-actinin-4 provides structural support to these microfilaments via its crosslinking and bundling activities, while linking them to components of the slit diaphragm and sites of adhesion. The gain affinity mutations in FSGS-associated *α*-actinin-4 substantially alter the properties of the actin cytoskeleton, rendering it more rigid and less dynamic. Therefore, the underlying pathogenesis of *ACTN4*-associated podocyte injury, glomerular filtration barrier dysfunction and the appearance of FSGS lesions are at least partly attributable to an aberrantly high interaction of *α*-actinin-4 with F-actin and its impact upon the cytoskeleton. 

In addition, the enhanced actin-*α*-actinin-4 interaction generates misfolded protein/aggregates, which could provide a parallel mechanism of podocyte dysfunction. As discussed above, misfolded proteins may induce dysfunction of the ubiquitin-proteasome system, that is, the misfolded proteins “choke” or “gum up” the proteasome, and this process may enhance proapoptotic stress in cellular compartments, including the ER. In addition, since ubiquitination regulates many essential cellular processes, including normal protein degradation, cell cycle, transcription, DNA repair, and protein trafficking, a disrupted ubiquitin-proteasome system may have broader adverse consequences for cell function [46]. Thus, the pathogenesis of FSGS associated with *α*-actinin-4 K256E may resemble processes in certain age-related or neurodegenerative diseases, where signs of ER stress, UPS dysfunction, and protein misfolding are observed [30, 45, 54, 56–58]. For example, in Huntington's disease, the expansion of a glutamine stretch within the N-terminal region of huntingtin gene generates a protein with severe neurotoxic properties. Expression of mutant huntingtin leads to a pronounced defect in ERAD, and UPR activation was noted in postmortem Huntington's disease brains. Familial amyotrophic lateral sclerosis has been linked to mutations in the gene encoding superoxide dismutase-1, and these mutations induce misfolding and aggregation of this protein, which is believed to contribute to neuronal dysfunction and death. Activation of the UPR is observed in mutant superoxide dismutase-1 transgenic mice, and increased levels of ER stress markers, as well as wild-type superoxide dismutase-1 aggregates have been reported in spinal cord tissue of sporadic amyotrophic lateral sclerosis. Modulation of ER stress pathways protected mice with experimental amyotrophic lateral sclerosis from disease progression. Other examples of neurodegenerative diseases associated with protein misfolding/aggregation and ER stress are Alzheimer's, Parkinson's, and prion diseases. Protein misfolding and UPS dysfunction also appears to play a role in desmin-related cardiomyopathies, which result in congestive heart failure [46]. By analogy to experimental neurodegenerative disease models, one must, however, be cautious in extrapolating the cell culture events, which are based on overexpression and a relatively brief experimental time frame, to a disease process that in humans takes many years to become established. Finally, the shuttling of the wild type, but not mutant *α*-actinin-4 between the cytoplasm and the nucleus, as well as the potential for disruption of gene regulation in the presence of mutant *α*-actinin-4, will require additional consideration as a potential mechanism of podocyte injury in FSGS. These various mechanistic relationships between abnormal proteins and cell injury will require further study.
